# *Helicobacter pylori* Infection Is Associated with Decreased Expression of SLC5A8, a Cancer Suppressor Gene, in Young Children

**DOI:** 10.3389/fcimb.2016.00121

**Published:** 2016-10-10

**Authors:** Andrea Orellana-Manzano, Miguel G. O'Ryan, Anne J. Lagomarcino, Sergio George, Mindy S. Muñoz, Nora Mamani, Carolina A. Serrano, Paul R. Harris, Octavio Ramilo, Asunción Mejías, Juan P. Torres, Yalda Lucero, Andrew F. G. Quest

**Affiliations:** ^1^Host-Pathogen Interaction Laboratory, Microbiology and Mycology Program, Faculty of Medicine, University of ChileSantiago, Chile; ^2^Center for Molecular Studies of the Cell (CEMC), Faculty of Medicine, University of ChileSantiago, Chile; ^3^Advanced Center for Chronic Diseases (ACCDiS), Faculty of Medicine, University of ChileSantiago, Chile; ^4^Computational Systems Biology Laboratory, Faculty of Pharmaceutical Sciences, University of São PauloSão Paulo, Brazil; ^5^Pediatric Gastroenterology and Nutrition Department, School of Medicine, Pontifical Catholic University of ChileSantiago, Chile; ^6^Center for Vaccines and Immunity, The Research Institute at Nationwide Children's HospitalColumbus, OH, USA; ^7^Department of Pediatrics and Pediatric Surgery (Eastern Campus), Faculty of Medicine, Luis Calvo Mackenna Hospital, University of ChileSantiago, Chile

**Keywords:** *Helicobacter pylori*, persistent infection, children, SLC5A8, cancer suppressor gene, qPCR detection

## Abstract

**Background:** *Helicobacter pylori* infects half of the world's population and causes gastric cancer in a subset of infected adults. Previous blood microarray findings showed that apparently healthy children, persistently infected with *H. pylori* have differential gene expression compared to age-matched, non-infected children. SLC5A8, a cancer suppressor gene with decreased expression among infected children, was chosen for further study based on bioinformatics analysis.

**Methods:** A pilot study was conducted using specific qRT-PCR amplification of SLC5A8 in blood samples from *H. pylori* infected and non-infected children, followed by a larger, blinded, case-control study. We then analyzed gastric tissue from *H. pylori* infected and non-infected children undergoing endoscopy for clinical purposes.

**Results:** Demographics, clinical findings, and family history were similar between groups. SLC5A8 expression was decreased in infected vs. non-infected children in blood, 0.12 (IQR: 0–0.89) vs. 1.86 (IQR: 0–8.94, *P* = 0.002), and in gastric tissue, 0.08 (IQR: 0.04–0.15) vs. 1.88 (IQR: 0.55–2.56; *P* = 0.001). Children who were both stool positive and seropositive for *H. pylori* had the lowest SLC5A8 expression levels.

**Conclusions:** *H. pylori* infection is associated with suppression of SCL5A8, a cancer suppressor gene, in both blood and tissue samples from young children.

**Key Points:** Young children, persistently infected with *Helicobacter pylori* show decreased expression of SLC5A8 mRNA in both blood and tissue samples as compared to non-infected children.

## Introduction

*Helicobacter pylori* (*H. pylori*) is a Gram-negative bacillus that infects half of the world's population and causes gastric cancer in a subset of infected adults (Malaty, 2007). According to a “National Health Survey” performed in Chile in 2003, the *H. pylori* seroprevalence rate in adults 17 years of age and older is 73% (Minsal, 2004).

*H. pylori* can be acquired during the first year of life, especially in populations living in lower socioeconomic environments, although information on childhood infection is scarce (Daugule and Rowland, 2008; Jaime et al., 2013; O'Ryan et al., 2013, 2015). We previously reported that 20–25% of children under 5 years of age from a semi-rural area of Chile are persistently infected with *H. pylori*, based on prolonged detection of antigen in stools (O'Ryan et al., 2013, 2015). Most persistently infected children do not present signs or symptoms during early childhood; to date, only 2/50 children followed up until a median age of 5 years and 10 months (range: 2 years 6month–8 years10 momth) have presented abdominal complaints. Comparing whole blood microarray data from infected children with non-infected, age-matched controls we identified differential expression of nearly 100 genes (O'Ryan et al., 2015). Based on bioinformatics analysis and gene characterization, and specifically because of its association with cancer, we targeted the gene SLC5A8 for further study.

SLC5A8 is a monocarboxylate transporter located in several tissues and organs, including the gastrointestinal tract (Zhang et al., 2010; Brim et al., 2011). This gene encodes a transmembrane protein that facilitates entrance of butyrate, propionate, and other short-chain fatty acids into normal cells. Incorporation of butyrate into tumor cells of both gastric and colon cancers favors tumor suppression by inhibiting inflammation, promoting cell differentiation, stimulating cell cycle arrest, and inducing apoptosis (Elangovan et al., 2013; Park et al., 2013; Gurav et al., 2015). Ueno et al. demonstrated that SLC5A8 was downregulated in adults with gastric cancer (Ueno et al., 2004) and was silenced in ~60% of individuals with colorectal cancer (Keenan and Frizelle, 2014), and others have confirmed that it is downregulated in several cancer types, including stomach (Ueno et al., 2004; Ganapathy et al., 2008), colon (Li et al., 2003; Miyauchi et al., 2004; Thangaraju et al., 2008), thyroid (Ganapathy et al., 2005; Porra et al., 2005; Schagdarsurengin et al., 2006), and breast (Thangaraju et al., 2006, 2009; Coothankandaswamy et al., 2013; Elangovan et al., 2013). Silencing has also been observed in earlier stages of cancer, including, colonic adenomas and colonic-crypts (Li et al., 2003). Although decreased SLC5A8 expression has been reported among individuals with gastric cancer, it has not been directly associated with *H. pylori* infection in adults or in children with symptomatic or asymptomatic infection.

The aim of this study was to confirm and expand on our blood microarray findings by determining if SLC5A8 expression levels are decreased in asymptomatic as well as symptomatic children infected with *H. pylori* compared to non-infected children.

## Methods

### Bioinformatics selection of the gene SLC5A8

We previously performed microarray analysis on blood samples from persistently, transiently and non-*H. pylori* infected children (O'Ryan et al., 2015). Further analysis of persistent compared to non-infected children identified 97 differentially expressed genes (Supplementary Table 2). These 97 genes were then classified by biological function using DAVID tools and by function and associated disease using the IPA® system (Huang et al., 2009; Krämer et al., 2014).

**Figure 1:**
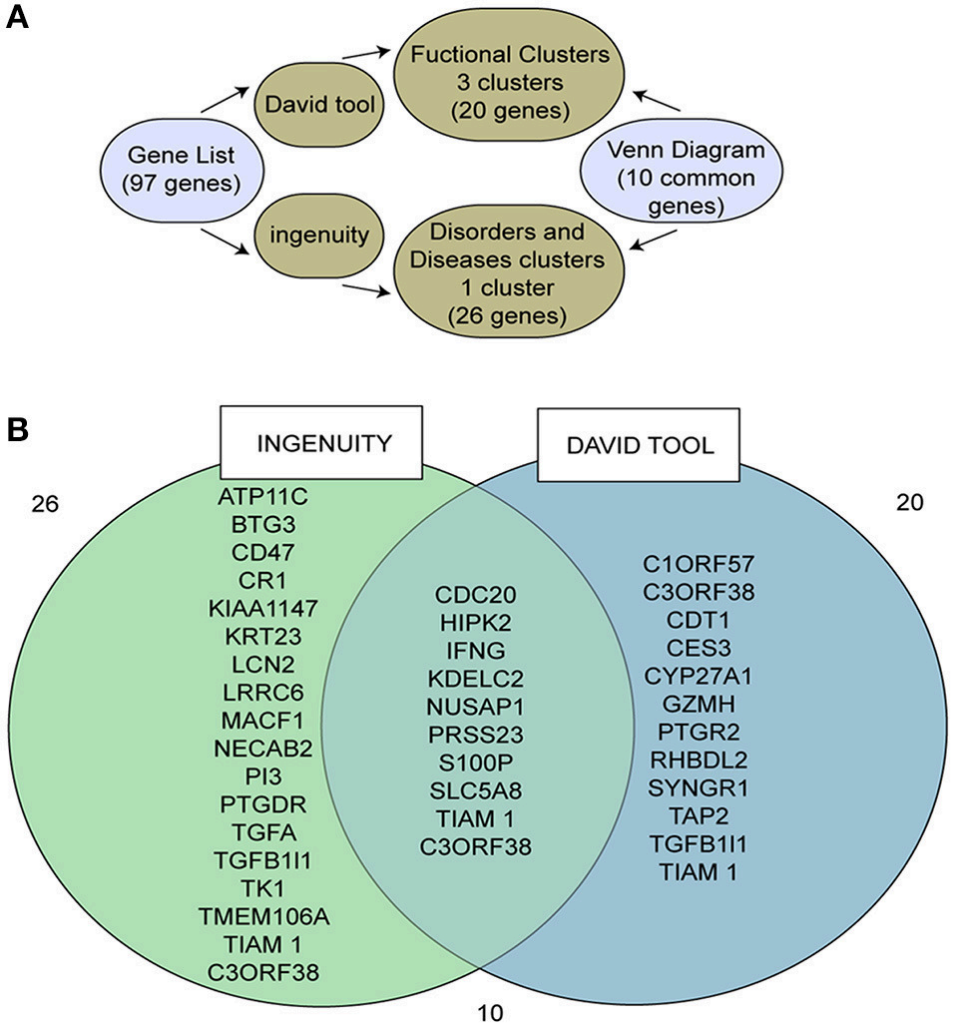
**Identification of the target gene, SLC5A8, using different bioinformatics tools. (A)** Scheme describing the bioinformatics pathway used to select our target gene; **(B)** Venn diagram of those genes identified by both Ingenuity and DAVID tools.

Based on analysis with DAVID tools, we selected 20 genes belonging to the three clusters with the highest scores (Figure 1, Supplementary Tables 3, 4). Additionally, 26 genes from one cluster associated with cancer were selected based on analysis with the IPA® system (Supplementary Table 5). Ten of these 36 genes overlapped (Figure 1B). A literature review focusing on cancer and *H. pylori* was performed for these 10 genes (see Supplementary Material for more details). This review indicated that eight genes are associated with cancer progression.

SLC5A8 was selected because it was recently identified as a cancer suppressor gene and because previous studies related downregulation of this gene to the progression of various types of cancer, including gastric (Ueno et al., 2004), colonic (Li et al., 2003; Thangaraju et al., 2008; Brim et al., 2011), thyroid (Porra et al., 2005), and breast cancers (Foglietta et al., 2014).

### Study design, recruitment, and *H. pylori* detection

Recruitment, blood sample, and data collection for the healthy child cohort as well as informed consent were approved by the Comité de Etica de Investigación, Servicio de Salud Metropolitano Norte and by the Comité de Etica, Universidad de Chile. Recruitment, tissue sample and data collection for children undergoing endoscopy was approved by the Comité de Etica de Investigación, Pontificia Universidad Católica (Project #PUC12-236). For the healthy child cohort, children were categorized as having a persistent *H. pylori* infection if they had three or more stool samples (obtained every 3–4 months) consecutively positive for *H. pylori* by ELISA over several years of follow up (median age of follow-up: 5 years 10 months, range: 2 years 6 month–8 years 10 month) (O'Ryan et al., 2013, 2015). Non-infected children were those whose stool samples were never positive for *H. pylori* by ELISA during the follow-up period.

*Blood samples H. pylori* persistently infected and non-infected children were matched by age, gender, and city. One milliliter of blood was obtained from all children by venipuncture, stored in Tempus blood RNA tubes (Thermo Fisher Scientific N°4342792) and transported to the laboratory following the appropriate cold chain protocols for gene expression assays.

**Figure 2:**
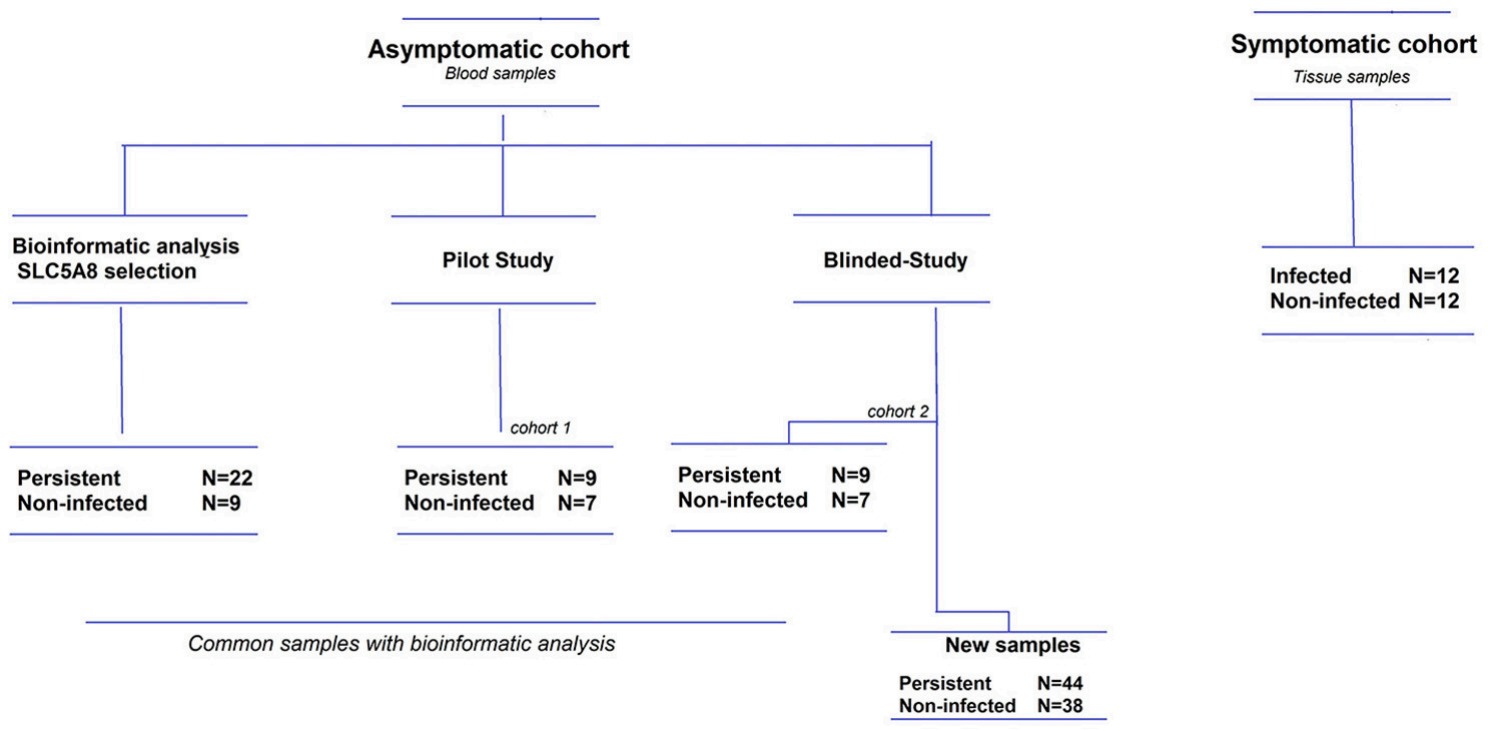
**Flow diagram of study patients**. The asymptomatic cohort is comprised of children under 5 years old, and the symptomatic cohort children under 12 years old. Some children from the asymptomatic cohort were included in both the bioinformatics analysis and the pilot/blinded-study, but all these samples were reprocessed within their respective studies.

We analyzed blood samples from children in our ongoing child cohorts, all of whom had been previously consented for a pilot study and a larger case-control study. For the pilot study, we randomly selected 16 blood samples from children under 5 years of age that had been previously analyzed by microarray (O'Ryan et al., 2015). For the blind, case-control study, 50 persistent and 44 non-infected children were selected (see Supplementary Material and Figure 2). The laboratory staff was blind to *H. pylori* infection status. *H. pylori* was detected by stool ELISA (O'Ryan et al., 2013, 2015) using the commercial ELISA Premier Platinum HpSA monoclonal antibody kit (Meridian Bioscience, Ohio, USA). All samples positive for 16S rRNA were tested for the presence of *CagA* by qRT-PCR, as described in the Supplementary Material.

*Stool samples* were obtained every 3 or 4 months and transported, following appropriate cold chain protocols, to our laboratory for detection of *H. pylori* antigen by ELISA. Stool samples were stored at −80°C. Bacterial DNA was extracted using the QIAamp DNA stool kit (Qiagen, Hilden, Germany) following manufacturer's instructions, with an additional bead-beating step to increase the yield of purified DNA. Detection of *16S* rRNA and *cagA* was performed using previously described primers (O'Ryan et al., 2015) (Supplementary Table 1). Amplification was performed in a reaction volume of 20 μL with Fast EvaGreen dye qPCR master mix (Biotium, Hayward. CA) with 4 μl of total purified DNA from each stool sample. The PCR reaction was performed using a STEP ONE thermocycler® (Applied Biosystems, Foster City, CA). A three-step PCR was used followed by melting curve analysis. Strain ATCC 43504 was used as the positive control. Amplification of total bacterial 16S rRNA gene was performed using an in-house PCR, in order to identify samples with possible PCR inhibitors. Each sample was analyzed in duplicate within each qRT-PCR reaction.

### *H. pylori* detection in gastric tissue

Gastric samples were analyzed from 24 patients, 5–12 years of age, who underwent endoscopy due to symptoms such as abdominal pain, vomiting, and/or diarrhea. All patients answered a clinical questionnaire that included age, gender, and relevant clinical data. Informed consent was obtained from the patient's parents or legal guardians. Upper gastrointestinal endoscopy was performed in the Hospital Clínico UC CHRISTUS and stored at −80°C in the Department of Pediatric Gastroenterology and Nutrition, School of Medicine, Pontificia Universidad Católica de Chile. Three biopsies were obtained from the gastric antrum, one for Rapid Urease Test (Hepytest®, BiosChile, Santiago, Chile), one for histological staining of *H. pylori* and one for mRNA extraction and SLC5A8 expression analysis. A patient was considered infected with *H. pylori* if both urease and histology staining were positive.

### *H. pylori* serology

A commercial ELISA kit for anti-*H. pylori* antibodies was used to analyze serum samples according to the manufacturer's instructions (Premier® *H. pylori;* Meridian Bioscience, Cincinnati, Ohio, USA), as previously described (O'Ryan et al., 2015).

### SLC5A8 gene expression assay in blood and tissue

RNA was extracted from blood using the QIAamp RNA Blood Mini Kit (ID: 52304, Qiagen, Hilden, Germany). It was then treated with DNases using the TURBO DNA-Free™ Kit (AM1907 AMBION, Thermo Fisher Scientific. Oklahoma, USA) and quantified using NanoQuant Take3 equipment. cDNA was obtained using the ImProm-II™ Reverse Transcription System (ID: A3800, Promega, Madison, Wisconsin, USA).

Endoscopic biopsies were frozen in liquid nitrogen immediately upon sampling and stored at −80°C until analysis. Total RNA was extracted using the RNAeasy kit (Qiagen; Hilden, Germany) and quantified using NanoQuant Take3 equipment. Reverse transcription was performed in a minimum of 1 μg total RNA; integrity was revised in 1% agarose gel using a commercial kit (AffinityScript QPCR cDNA synthesis kit, Agilent Technologies; Santa Clara, California, USA). A mix of DT oligos and random primers was used for the synthesis of cDNA.

Taqman® probes (Applied Biosystems, Foster City, California, USA) were used for the qPCR gene expression assay. Expression of SLC5A8 was normalized to the housekeeping gene β-actin and expression levels are reported using the 2^ΔCT^ quantification method (Rao et al., 2013). qRT-PCR assays were performed in a STEP ONE thermocycler® (Applied Biosystems, Foster City, CA). A gastric tissue sample that expressed SLC5A8 was used as a positive control; non-reverse transcription RNA and water were used as negative controls. All samples were analyzed in three independent experiments; in each experiment we ran the samples in duplicate (in order to obtain one valid value). The average of the values from the three experiments was used for analysis.

### Statistical analysis

Statistical analysis was performed using R Version 3.0.0 (R Development Core Team, 2013). For categorical variables, groups were compared using the Chi-squared test; the Kruskal–Wallis test was used for continuous, non-normal variables. *P*-values ≤ 0.05 were considered significant.

## Results

### Expression of SLC5A8 is decreased in blood samples from infected compared with non-infected children

#### Pilot study

**Figure 3:**
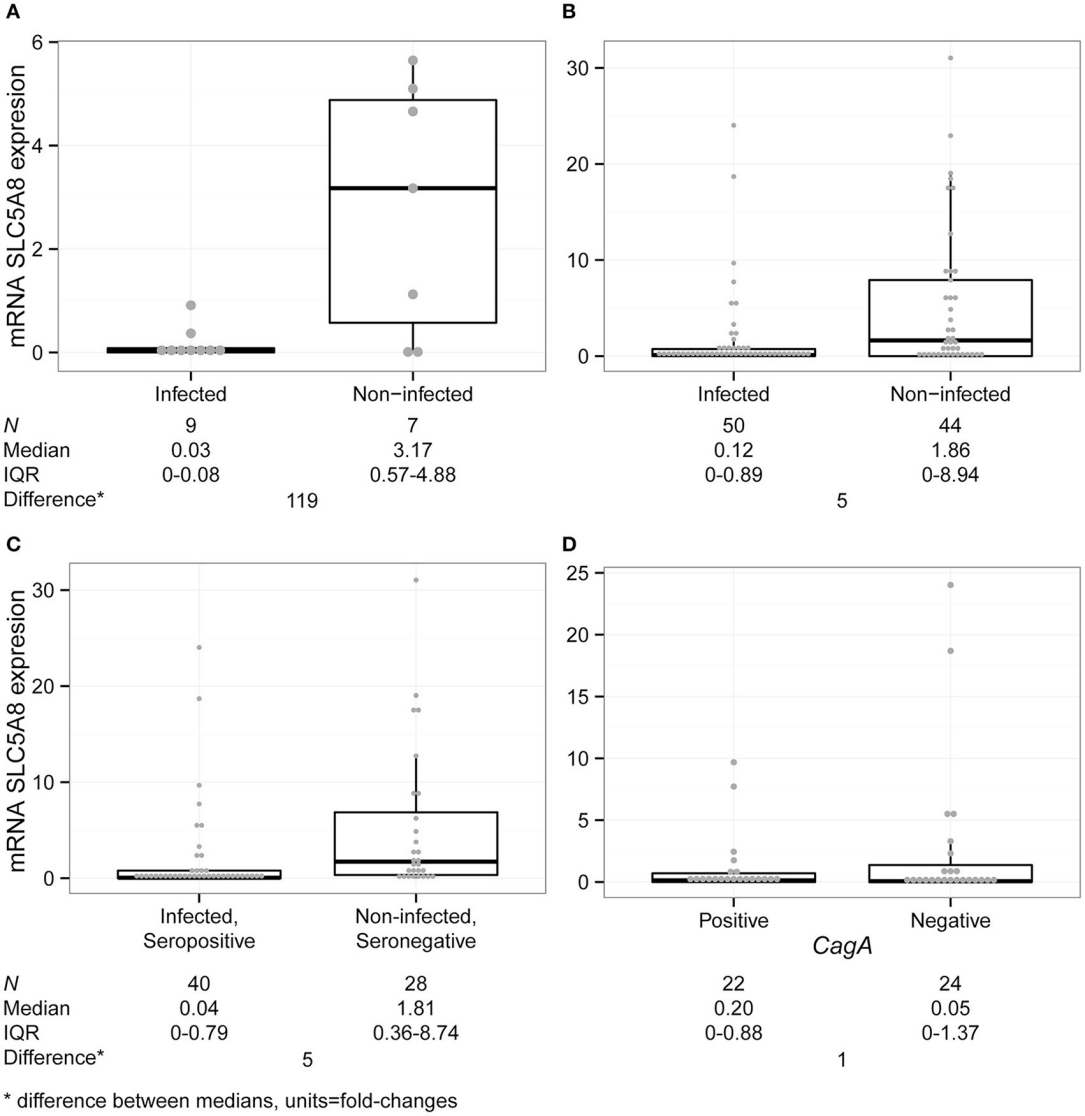
**SLC5A8 expression is decreased in blood samples from infected compared with non-infected children**. All four panels show a dot plot overlying a boxplot (showing the median and IQR; points outside the whisker lines are considered outliers, as specified by Turkey's Test). Summary statistics are shown below each panel. **(A)** mRNA SLC5A8 expression is significantly decreased in blood samples from *H. pylori* infected compared to non-infected children in the pilot study (Kruskal–Wallis test, *P* = 0.050). **(B)** In a blinded case-control study of SLC5A8 expression in blood samples, expression levels are significantly decreased in infected compared to non-infected children (Kruskal–Wallis test, *P* = 0.002). Four outliers were not shown for space (infected: 42; non-infected: 72, 212, 379). **(C)** mRNA SLC5A8 expression is decreased in infected and *H. pylori* seropositive children vs. non-infected, seronegative children (Kruskal–Wallis test, *P* = 0.002). One outlier was not shown for space (non-infected/seronegative: 378.51) **(D)** mRNA SLC5A8 expression does not differ (based on the Kruskal–Wallis test) between CagA positive and negative children. One outlier was not shown for space (CagA positive: 42.03).

We performed quantitative reverse transcription PCR (qRT-PCR) for SLC5A8 and β-Actin on blood samples from nine infected and seven non-infected children (for definitions see Supplementary Material). Overall, persistently infected children had lower levels of SLC5A8 mRNA (0.03; IQR 0–0.08), compared to non-infected children (3.17; IQR 0.57–4.88; *P* = 0.050; Figure 3A). Similarly, we compared the median expression of each group observing an approximately 120-fold decrease in expression in infected children compared with non-infected children.

#### Blinded case-control study of SLC5A8 expression in blood samples

We blindly analyzed 94 blood samples, 50 from *H. pylori* infected and 44 from non-infected children. There were no statistically significant differences between infected and non-infected children in demographic variables, family and past medical history, medication use and gastric symptomatology (Table 1). SLC5A8 expression was decreased in the infected (0.12; IQR 0–0.89) compared to the non-infected group (1.86; IQR 0–8.94; *P* = 0.002; Figure 3B). Similarly, a significant difference was observed when we categorized expression levels using a cutoff of < 2 (< 2 and >1) and (≤ 1) (Table 2; *P* = 0.008). The comparison of the median expression levels of each group revealed a 5-fold decrease in infected vs. non-infected children.

**Table 1:** **Demographic, clinical, and family characteristics of children enrolled in the case-control study according to *H. pylori* status**.

	***H. pylori status***	
	**Non-infected**	**Infected**
Characteristic, *N* (%)^A^	*N* = 44	*N* = 50
Male	25 (57)	29 (58)
Age (months), median (IQR)	63 (25–91)	63 (24–99)
Attended day care prior to age 2	13 (30)	16 (32)
Attended day care prior to age 4	13 (30)	10 (20)
Treated with Omeprazole	0/41 (0)	1/46 (2)
Family history of *H. pylori*	7 (16)	3 (6)
Family history of gastric cancer	9 (20)	6 (12)
Family history of gastric ulcer	13 (30)	15 (30)

*None of the listed characteristics were significantly different between the two groups*.

A *unless otherwise noted*.

**Table 2:** **Distribution of SLC5A8 expression levels in *H. pylori* infected and non-infected children**.

**Number (%) of subjects with indicated expression level according to *H. pylori* infection status**		
	**Non-infected**	**Infected**
Expression level	*N* = 44	*N* = 50
> 2	26 (59)	12 (24)
≤ 2 and >1	6 (14)	19 (38)
≤ 1	12 (27)	19 (38)

*P = 0.008 by Chi-squared test*.

It is worth also mentioning that there were samples in which expression levels were not detectable, 12 infected and 4 non-infected children. We decided to include these samples as part of the analysis, assigning them an expression value of 0. However, even when excluding these samples from the analysis the relationship remains significant (non-infected: 2.92, IQR 0.72–10.84 vs. infected: 0.44, IQR 0.03–2.17: *P* = 0.005).

In addition to stool antigen detection, we compared mRNA SLC5A8 in a subset of children with differing anti-*H. pylori* serology status. Out of 50 stool ELISA positive children, 40 were seropositive for *H. pylori* (80%), whereas 4/33 (12%) stool ELISA negative children were seropositive (*P* < 0.001; 11 non-infected children do not have serology results available). For *H. pylori* infected seropositive children median SLC5A8 expression levels were 0.04 (IQR: 0–0.79) vs. 1.81 (IQR: 0.36–8.74) for non-infected seronegative children (*P* = 0.002; Figure 3C). A comparison of the median expression levels revealed a 5-fold decrease in infected/seropositive compared to non-infected/seronegative children.

Real time PCR (qRT-PCR) 16SrRNA amplification was performed on one stool sample per persistent child in order to confirm ELISA positive results; 46/50 samples were confirmed. These samples were then analyzed by qRT-PCR for the *CagA* virulence gene. No significant difference in SLC5A8 expression was observed between *CagA* positive, (*n* = 22, 0.20; IQR: 0–0.88) or negative children (*n* = 24, 0.05 IQR: 0–1.37; *P* = NS) suggesting the lack of a consistent relationship between SLC5A8 expression and infection with a *CagA* positive or negative *H. pylori* strains (Figure 3D).

### SLC5A8 expression is decreased in tissue samples of *H. pylori* infected compared to non-infected children

#### Blinded case-control study of SLC5A8 expression in gastric biopsies of H. pylori infected and non-infected children

**Figure 4:**
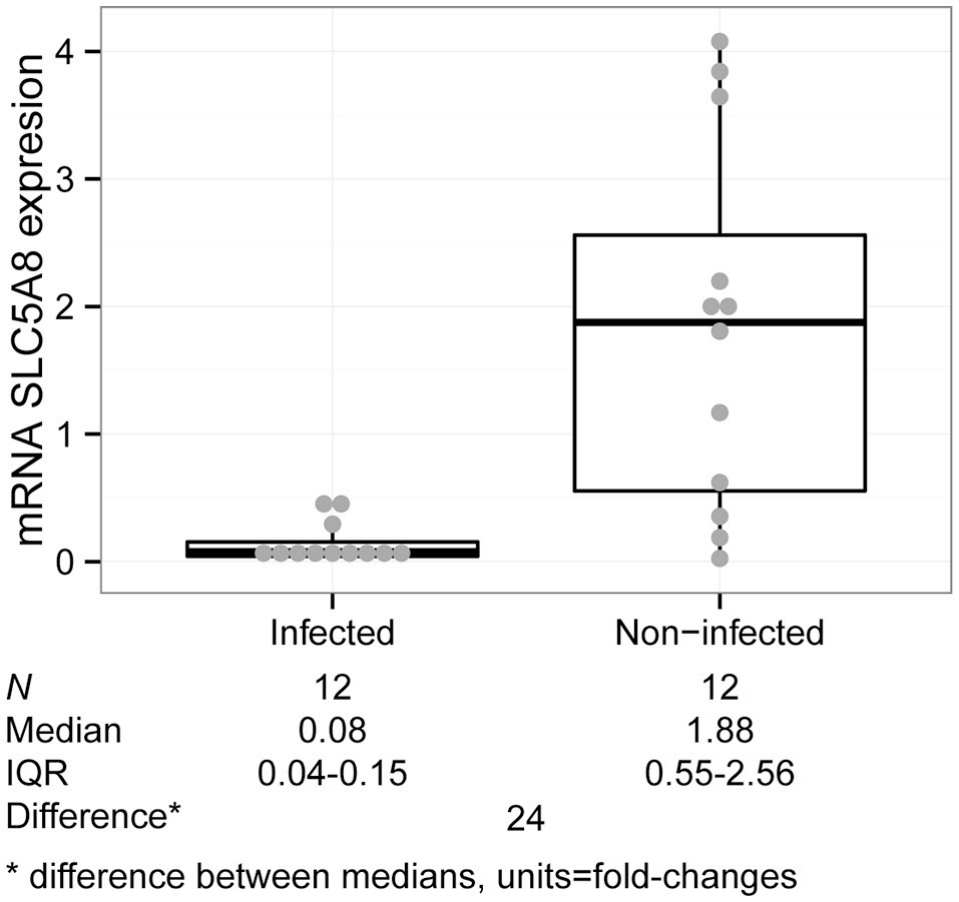
**SLC5A8 expression is decreased in *H. pylori* infected compared to non-infected children in gastric tissue samples (Kruskal–Wallis test, *P* = 0.001)**. The plot is composed of a dot plot overlying a boxplot (showing the median and IQR; points outside the whisker lines are considered outliers, as specified by Turkey's Test); summary statistics are shown beneath the graph.

We analyzed gastric tissue from 12 *H. pylori* infected (as determined by positive rapid urease test and histology staining) and 12 non-infected children under 10 years of age who underwent endoscopy due to abdominal complaints. There were no significant differences in demographic characteristics or gastric symptomatology between groups (Table 3). SLC5A8 mRNA was significantly lower in infected (0.08; IQR: 0.04–0.15) compared to non-infected children (1.88; IQR: 0.55–2.56; *P* = 0.001; Figure 4). The difference in the median expression of each group revealed a 24-fold decrease among infected compared to non-infected children.

**Table 3:** **Demographic, symptomatology, and gastric biopsy results of infected and non-infected children undergoing endoscopy**.

	***H. pylori* status**		
	**Non-infected**	**Infected**	***P*-value**
Characteristic, *N* (%)	*N* = 12	*N* = 12	
Male	7 (29)	5 (21)	NS
Age in years, median (IQR)	12.5 (5–17)	12 (8–14)	NS
Body Mass Index	20.1 (16.2–23.9)	21.3 (13.9–31.2)	NS
**SYMPTOM SEVERITY**			
No symptom	0	1	NS
Mild	4 (33)	1	NS
Moderate	5 (42)	4 (33)	NS
Severe	3 (25)	6 (50)	NS
**SYMPTOMS DURATION**			
1 week	7 (58)	5 (46)	NS
1 month	3 (25)	4 (36)	NS
1 year	2 (17)	2 (18)	NS
**UPPER GI ENDOSCOPY REPORT**			
Normal	5 (42)	0	0.016
Gastropathy	3 (25)	0	0.064
Nodules	4 (33)	12 (100)	< 0.001
HISTOLOGY REPORT^*^			
*Gastritis*			
Absence	6 (55)	0	NS
Mild	5 (45)	5 (55)	NS
Moderate	0	4 (45)	0.011
*Inflammatory activity*			
Absence	11 (100)	4 (45)	NS
Presence	0	5 (55)	0.08
*Lymphoid follicles*			
Presence	0	2 (22)	NS
Absence	11 (100)	7 (78)	NS
*Atrophy*			
Presence	1	0	NS
Absence	10 (91)	9 (100)	NS

* *data available for 20 subjects*.

Histological information was available in 20 of the 24 children with tissue samples. A pathologist determined the degree of gastric damage, inflammatory activity, presence of lymphoid follicles, and the presence or absence of gastric atrophy (Supplementary Table 6). The higher the score, the greater the gastric damage, and inflammatory processes; the score is based on a scale of 0 (no damage), 1 (mild damage), 2 (moderate damage) to 3 (severe damage). Symptoms, such as vomiting, recurrent abdominal pain, were also scored by the treating physicians as low, moderate and severe. Information on symptom duration was grossly classified as occurring only during the previous week, for at least a month, or for at least 1 year.

We analyzed each indicator of gastric damage and symptomatology (inflammatory activity, gastritis, lymphoid follicles, atrophy, symptoms and duration of symptoms). More severe gastritis (*P* = 0.019), and presence of lymphoid follicles (*P* = 0.044) were significantly associated with decreased SLC5A8 expression levels (Supplementary Figures 1, 2). The following variables did not show a significant association with expression levels: Inflammatory activity (*P* = 0.150), atrophy (*P* = 0.603), symptoms (*P* = 0.872) (Supplementary Figures 3–5) and symptom duration (*P* = 0.505).

Importantly, SLC5A8 expression levels were significantly lower among infected children compared to non-infected with similar conditions of inflammatory activity, lymphoid follicles, and atrophy. Expression was lower for similar levels of gastritis, but differences were not significant (Table 4).

**Table 4:** **mRNA SLC5A8 expression in 11 infected and 9 non-infected children according to levels of histological damage**.

	**Median (IQR) of indicated variable by *H. pylori status***				
		**Infected**		**Non-infected**	***P*-value**
*Gastritis*	*(N)*		*(N)*		
Normal	0	NS	6	1.80 (1.21–2.19)	
Mild	5	0.29 (0.20–0.45)	5	1.94 (1.06–3.74)	0.34
Moderate	4	0.07 (0,02–0.07)	0	NS	
Severe	0	NS	0	NS	
*Inflammatory activity*					
Presence	5	0.10 (0.09–0.37)	0	NS	
Absence	4	0.06 (0.02–0.10)	11	2.00 (0.35–3.64)	0.03
*Lymphoid Follicles*					
Presence	2	0.0254 (0.025–0.026)	0	NS	
Absence	7	0,20 (0,09–0,45)	11	1.88 (0.55–2.60)	0.016
*Atrophy*					
Presence	0	NS	1	1,81	
Absence	9	0.10 (0.06–0.29)	10	1.94 (0.35–3.74)	0.01

*+NS: No subjects in this category*.

## Discussion

In two separate evaluations, a pilot study and a case-control study, median SLC5A8 expression levels were decreased in blood samples from *H. pylori* infected children less than 6 years of age as compared to non-infected children. This finding was also confirmed in gastric tissue of infected compared to non-infected children. *H. pylori* modulation of gene expression has been demonstrated previously in adult populations (Hanada and Graham, 2014; Obayashi et al., 2015); however, this is the first report of a significant association between SLC5A8 gene expression and *H. pylori* infection in both symptomatic and asymptomatic children.

The fact that SLC5A8 expression is reduced in fresh whole blood samples (which include several components such as plasma, red blood cells, white blood cells, and platelets) from *H. pylori* infected children raises several hypotheses as to how an infection occurring at the gastric level is affecting the circulatory system. Extracellular vesicles (EVs), which carry mRNAs, noncoding RNA and proteins generated from different biological processes may play a role (EL Andaloussi et al., 2013; Vader et al., 2014). EVs are membrane-enclosed structures that are released into the surrounding environment by nearly all cell types. After separation from the plasma membrane, these vesicles may travel from the extracellular/intracellular space of gastric epithelial cells into the blood stream (Vader et al., 2014). A second hypothesis is that lymphocytes, neutrophils, and macrophages present during an epithelial inflammatory event can be detected in circulating blood (Cadamuro et al., 2014). This would require a peripheral inflammatory environment during chronic *H. pylori* infection, including the presence of macrophages capable of storing genetic information detectable in blood (Fehlings et al., 2012).

Our findings pose several questions and open several areas for future research. Reduced SLC5A8 expression associated with *H. pylori* infection at such an early age may be sustained over time or may be transitory or fluctuating in association with various factors. There was no correlation between expression levels and a child's age (data not shown). Expectedly, *H. pylori* was associated with more severe gastric tissue damage, and more severe damage (gastritis severity and presence of lymphoid follicles) was associated with decreased SLC5A8 expression levels. Importantly although, in children with similar levels of gastric damage, infection was significantly associated with decreased expression, strongly suggesting that infection is the key player in this effect. Of note is that expression is reduced among infected vs. non-infected children overall, but individually many non-infected children had very low expression levels. Whether infection plays a role in gene expression in only a subset of children, and if these children are possibly at higher risk for cancer occurrence decades later will require long-term cohort monitoring and/or cross-sectional studies in different age groups.

We did not find an association between SLC5A8 expression levels and the presence of *CagA* among infected individuals. *CagA* amplification in stools is difficult (O'Ryan et al., 2013), and although each sample was tested repeatedly before attributing a positive or negative status, there remains some degree of uncertainty based on the fact that we were attempting to detect a virulence factor present in *H. pylori* at the gastric level in stool samples. Obtaining the *H. pylori* strain directly from the stomach would be ideal, but ethically not feasible for apparently healthy children; unfortunately, there has been little success in culturing this bacteria from stool samples (O'Ryan et al., 2013, 2015). In addition, it is possible that persistent infections may be caused by different strains over time, making our results, based on one sample, inconclusive.

At this point, it might be interesting to speculate on how reduced SLC5A8 expression in host cells may benefit *H. pylori*. Indeed, this pathogen has been shown to modulate host gene expression in ways that favor host infection (Matsushima et al., 2011; Kim et al., 2012; Hanada and Graham, 2014; O'Ryan et al., 2015; Rossi et al., 2016). As mentioned in this manuscript, SLC5A8 is implicated in the control of short-chain fatty acid uptake, which in turn modulates the expression of cell cycle control and pro-inflammatory genes. Potentially, therefore, one may speculate that loss of SLC5A8 expression induced by *H. pylori* should promote cell proliferation and inflammation, which in turn could favor the infection process and enhance severity of the infection.

There are limitations to this study. The number of tissue samples analyzed is relatively small and we were not able to analyze blood and tissue from the same children due to lack of dual samples. For healthy children who provided blood samples, it was not ethical to request an endoscopy and tissue biopsy due to their lack of symptoms; for children providing a tissue sample due to symptoms as part of another study, collection of a blood sample was not contemplated at the time of original study design. Histological information was unavailable for 4 children providing gastric biopsy samples, and the standardized Sydney Score was not initially considered, so a less conventional evaluation of gastric damage severity was used. Lastly, we only analyzed one of the genes identified by gene wide array; analysis of additional genes in light of our findings, represent a new challenge.

Future prospective studies should focus on culturing *H. pylori* strains in both children and adults requiring endoscopy, with different stages of gastric pathology, in order to confirm differences in SLC5A8 expression levels. In addition, longitudinal studies of infected children, including gene expression levels over time, will be required to determine the co-occurrence of persistent infection and decreased gene expression levels. Importantly, studies using *in vitro* models, including different cell lines, will be required to understand the mechanisms involved in reduced gene expression.

## Author contributions

AO conceived, designed, analyzed data, coordinated the study and wrote the manuscript with substantial support from MO, AL, and AQ. SG designed the research experimental studies in stool samples. MO, NM, YL, and JT designed the cohort studies. MM designed the bioinformatics analysis with support from OR and AM. CS and PH designed and performed the clinical studies in children requiring endoscopy, submitting the tissue samples. All authors reviewed and approved the manuscript.

## Funding

This work was supported by the Fondo Nacional de Desarrollo Científico y Tecnológico [1061079 and 1100514 to MO, 1130387 to PH, and 11140232 to CS]; Comisión Nacional de Investigación Científica y Tecnológica [21140520 to AO]; Fondo de Financiamiento de Centros de Investigación en Áreas Prioritarias [15130011 to AQ] and Secretaría de Educación Superior, Ciencia, Tecnología e Innovación [to AO].

### Conflict of interest statement

The authors declare that the research was conducted in the absence of any commercial or financial relationships that could be construed as a potential conflict of interest.
