# Supplementary material for: Helicobacter pylori Infection Is Associated with Decreased Expression of SLC5A8, a Cancer Suppressor Gene, in Young Children
Source: Front Cell Infect Microbiol. 2016 Oct 10;6:121. doi: 10.3389/fcimb.2016.00121 (PMC5056170; doi:10.3389/fcimb.2016.00121)

**Supplementary Table 1. Primers for qRT-PCR used to identify *H. pylori* and *cagA*.**

| *Gene* | *Sequence 5'--------3'* | *Amplicon size (bp)* | *NCBI Accession Number* |
| --- | --- | --- | --- |
| *16S RNA* | Forward: CTGGAGAGACTAAGCCCTCC | 109 | AE000511 |
|  | Reverse: ATTACTGACGCTGATTGTGC |  |  |
| *cagA* | Forward: GATCAAAAGTTCATGGGCGTG | 189 | L11714 |
|  | Reverse: GGCTATATCTGGTTGGACATGG |  |  |

**Supplementary Table 2. Differentially expressed genes between *H. pylori*-infected and non-infected children based on microarray analysis of whole blood samples.** Microarray analysis was conducted on samples from 9 non-infected and 44 infected children (22 persistent, 22 transitory), using Illumina Human HT12 V4 beadchips and scanned on the Illumina Bead-station 500. The table lists all genes found to be significantly different between non-infected versus infected children (based on Kruskal–Wallis, with a significance level of *P* ≤0.05).

| *Systematic* | *Common* | *Synonyms* | *Genbank* |
| --- | --- | --- | --- |
| ILMN_2224486 | C3ORF14 | HT021 | NM_020685.3 |
| ILMN_1682993 | NKG7 | GIG1 | NM_005601.3 |
| ILMN_1900998 | HS.513000 |  | BC039397 |
| ILMN_1651237 | CDT1 | DUP; RIS2 | NM_030928.2 |
| ILMN_2389876 | TGFB1I1 | TSC-5; ARA55; HIC5; HIC-5 | NM_015927.3 |
| ILMN_3218292 | LOC202781 |  | XM_001719263.1 |
| ILMN_2073184 | S1PR5 | SPPR-2; SPPR-1; Edg-8; S1P5; S1PR5 | NM_030760.3 |
| ILMN_2105253 | PTGR2 | FLJ39091; PGR-2; PGR2; DKFZp686P10120 | NM_152444.1 |
| ILMN_1663437 | NECAB2 | EFCBP2; NECAB2 | NM_019065.2 |
| ILMN_1806037 | TK1 | TK2 | NM_003258.2 |
| ILMN_3274543 | LOC286002 |  | XM_001715026.1 |
| ILMN_3267800 | LOC100130276 | | XM_001722797.1 |
| ILMN_3249244 | TMEM106A | MGC20235 | NM_145041.1 |
| ILMN_1704985 | CYP27A1 | CYP27; CTX; CP27 | NM_000784.2 |
| ILMN_2053536 | RHBDL2 | RRP2; MGC16997 | NM_017821.3 |
| ILMN_1687519 | SNAP23 | SNAP23A; SNAP23B; HsT17016 | NM_003825.2 |
| ILMN_3187403 | LOC100129905 | | XM_001726007.1 |
| ILMN_1795063 | ZADH2 | MGC45594 | NM_175907.3 |
| ILMN_1811221 | SLC5A8 | SMCT; MGC125354; AIT | NM_145913.2 |
| ILMN_2073543 | C15ORF63 | HSPC136 | NM_016400.2 |
| ILMN_1695435 | LOC653610 |  | XM_928387.1 |
| ILMN_2409298 | NUSAP1 | LNP; FLJ13421; SAPL; BM037; PRO0310p1; Q0310; ANKT | NM_018454.5 |
| ILMN_1777499 | LOC731007 |  | XM_001132080.1 |
| ILMN_2270100 | C6ORF204 | MGC131785; RP11-57K17.2; bA57K17.2; NY-BR-15 | NM_001042475.1 |
| ILMN_1797776 | PRSS23 | MGC5107; SPUVE; SIG13; ZSIG13 | NM_007173.4 |
| ILMN_1687440 | HIPK2 | PRO0593 | NM_022740.2 |
| ILMN_1671482 | GALM | BLOCK 25 | NM_138801.1 |
| ILMN_3213185 | LOC645452 |  | XM_939733.2 |
| ILMN_2313889 | ZNF682 | FLJ90362; BC39498_3 | NM_033196.2 |
| ILMN_1758371 | IL1R2 | IL1RB; CD121b; MGC47725 | NM_004633.3 |
| ILMN_1663390 | CDC20 | CDC20A; bA276H19.3; MGC102824; p55CDC | NM_001255.2 |
| ILMN_1657446 | C1ORF57 | RP4-659I19.2; RP4-678E16.2; MGC13186; FLJ11383 | NM_032324.1 |
| ILMN_1791593 | DENND5B | FLJ41648; DKFZp686P1174; FLJ43333 | NM_144973.3 |
| ILMN_2062714 | PTGDR | MGC49004; ASRT1; DP; AS1 | NM_000953.2 |
| ILMN_1697742 | C3ORF38 | MGC26717 | NM_173824.2 |
| ILMN_1667330 | LOC113386 | FLJ44718; FLJ46452 | NM_138781.2 |
| ILMN_2346137 | ZNF557 | MGC4054 | NM_001044387.1 |
| ILMN_1863233 | HS.374420 |  | CR613022 |
| ILMN_1660549 | GPR177 | C1orf139; FLJ23091; WLS; EVI; MGC131760; MRP; MGC14878 | NM_001002292.1 |
| ILMN_1804611 | C12ORF23 | FLJ13959; MGC17943; FLJ11721 | NM_152261.1 |
| ILMN_2207291 | IFNG | IFI; IFG | NM_000619.2 |
| ILMN_2063114 | TAF1D | MGC5306 | NM_024116.2 |
| ILMN_3297130 | LOC729402 |  | NM_001139505.1 |
| ILMN_1678238 | ZNF683 | MGC33414; RP11-569G9.6 | NM_173574.2 |
| ILMN_1742601 | CR1 | C3BR; CD35; KN | NM_000651.4 |
| ILMN_1737935 | MACF1 | KIAA0465; FLJ45612; ACF7; ABP620; KIAA1251; OFC4; MACF; FLJ46776 | NM_012090.3 |
| ILMN_1693192 | PI3 | SKALP; ESI; WAP3; WFDC14; MGC13613 | NM_002638.2 |
| ILMN_1651752 | CXORF21 | FLJ11577 | NM_025159.1 |
| ILMN_3239653 | RAX2 | QRX; MGC15631; ARMD6; CORD11; RAXL1 | NM_032753.3 |
| ILMN_3302919 | MYOF |  | NM_013451.3 |
| ILMN_2074762 | FCRL6 | FLJ16056; FcRH6 | NM_001004310.1 |
| ILMN_1651557 | KDELC2 | MGC33424 | NM_153705.4 |
| ILMN_1690237 | PCDHB19P | PCDHB19; PCDH-PSI5 | NR_001282.1 |
| ILMN_1655702 | ABHD5 | MGC8731; CGI58; CDS; NCIE2; IECN2 | NM_016006.3 |
| ILMN_2165975 | CES3 | ES31; FLJ21736; Br3 | NM_024922.3 |
| ILMN_1731233 | GZMH | CTSGL2; CCP-X; CGL-2; CTLA1; CSP-C | NM_033423.3 |
| ILMN_1771482 | KIAA1324 | MGC150624; RP11-352P4.1; EIG121 | NM_020775.2 |
| ILMN_1692223 | LCN2 | NGAL | NM_005564.3 |
| ILMN_1791545 | KRT23 | MGC26158; CK23; DKFZP434G032; K23; HAIK1 | NM_015515.3 |
| ILMN_3211746 | LOC100131166 | | XR_039303.1 |
| ILMN_1721712 | SYNGR1 | MGC:1939 | NM_145731.2 |
| ILMN_1680692 | NUCKS1 | NUCKS; FLJ21480; JC7 | NM_022731.2 |
| ILMN_2356991 | CD47 | OA3; MER6; IAP | NM_001777.3 |
| ILMN_3245116 | GOLIM4 | P138; GIMPC; GOLPH4; GPP130 | NM_014498.3 |
| ILMN_3222998 | LOC729513 |  | XR_039881.1 |
| ILMN_2154836 | BTG3 | TOB55; TOB5; MGC8928; ANA; TOFA | NM_006806.3 |
| ILMN_1738549 | VPREB1 | IGI; IGVPB; VPREB | NM_007128.2 |
| ILMN_1754249 | LOC442535 |  | NM_001013738.1 |
| ILMN_1700307 | FLJ38969 |  | XM_928774.1 |
| ILMN_1728603 | LOC643713 |  | XM_927009.1 |
| ILMN_1885728 | KIAA1147 |  | XM_001130020.1 |
| ILMN_1801216 | S100P | MIG9 | NM_005980.2 |
| ILMN_3210741 | LOC642956 |  | XM_938166.3 |
| ILMN_1759250 | TAP2 | RING11; PSF2; APT2; ABC18; ABCB3; D6S217E | NM_018833.2 |
| ILMN_1655577 | TIAM1 | FLJ36302 | NM_003253.2 |
| ILMN_1779353 | PUS7 | FLJ20485; MGC17720; KIAA1897 | NM_019042.3 |
| ILMN_1793572 | C21ORF81 |  | NM_153750.1 |
| ILMN_1805175 | TGFA | TFGA | NM_001099691.1 |
| ILMN_1845086 | HS.193784 |  | AL110204 |
| ILMN_1739683 | LRRC6 | LRTP; TSLRP | NM_012472.3 |
| ILMN_1770818 | LOC642342 |  | XM_925876.1 |
| ILMN_1702636 | TUBB6 | MGC132410; HsT1601; MGC4083; TUBB-5 | NM_032525.1 |
| ILMN_1730454 | FOLR3 | FR-gamma; gamma-hFR; FR-G | NM_000804.2 |
| ILMN_3285785 | LOC647307 |  | XR_039752.1 |
| ILMN_2389810 | ATP11C | ATPIQ; ATPIG | NM_173694.3 |

**Supplementary Table 3.** **Gene clusters according to DAVID tools database.** Genes were clustered based on function; each cluster represents one group of genes. Clusters were ranked by enrichment score. The Group Enrichment Score, the geometric mean (in -log scale) of a member's *P*-values in a corresponding annotation cluster, is used to rank their biological significance.

| *Annotation Cluster 1* | | | | |
| --- | --- | --- | --- | --- |
| *Enrichment Score: 1.295* | | | | |
| Category | Term | Count | *P*-value | Genes |
| GOTERM_MF_FAT | GO:0004252~serine-type endopeptidase activity | 4 | 0.011 | ILMN_1731233. ILMN_2053536. ILMN_1657446. ILMN_1797776 |
| GOTERM_MF_FAT | GO:0008236~serine-type peptidase activity | 4 | 0.016 | ILMN_1731233. ILMN_2053536. ILMN_1657446. ILMN_1797776 |
| GOTERM_MF_FAT | GO:0017171~serine hydrolase activity | 4 | 0.017 | ILMN_1731233. ILMN_2053536. ILMN_1657446. ILMN_1797776 |
| GOTERM_MF_FAT | GO:0004175~endopeptidase activity | 4 | 0.102 | ILMN_1731233. ILMN_2053536. ILMN_1657446. ILMN_1797776 |
| GOTERM_MF_FAT | GO:0070011~peptidase activity. acting on L-amino acid peptides | 4 | 0.227 | ILMN_1731233. ILMN_2053536. ILMN_1657446. ILMN_1797776 |
| GOTERM_MF_FAT | GO:0008233~peptidase activity | 4 | 0.247 | ILMN_1731233. ILMN_2053536. ILMN_1657446. ILMN_1797776 |
| *Annotation Cluster 2* | | | | |
| *Enrichment Score: 1.280* | | | | |
| GOTERM_BP_FAT | GO:0006915~apoptosis | 6 | 0.038 | ILMN_1697742. ILMN_1731233. ILMN_1655577. ILMN_2207291. ILMN_1687440. ILMN_1811221 |
| GOTERM_BP_FAT | GO:0012501~programmed cell death | 6 | 0.040 | ILMN_1697742. ILMN_1731233. ILMN_1655577. ILMN_2207291. ILMN_1687440. ILMN_1811221 |
| GOTERM_BP_FAT | GO:0008219~cell death | 6 | 0.070 | ILMN_1697742. ILMN_1731233. ILMN_1655577. ILMN_2207291. ILMN_1687440. ILMN_1811221 |
| GOTERM_BP_FAT | GO:0016265~death | 6 | 0.072 | ILMN_1697742. ILMN_1731233. ILMN_1655577. ILMN_2207291. ILMN_1687440. ILMN_1811221 |
| *Annotation Cluster 3* | | | | |
| *Enrichment Score: 0.936* | | | | |
| GOTERM_BP_FAT | GO:0051969~regulation of transmission of nerve impulse | 3 | 0.076 | ILMN_1801216. ILMN_2207291. ILMN_1721712 |
| GOTERM_BP_FAT | GO:0031644~regulation of neurological system process | 3 | 0.082 | ILMN_1801216. ILMN_2207291. ILMN_1721712 |
| GOTERM_BP_FAT | GO:0044057~regulation of system process | 3 | 0.249 | ILMN_1801216. ILMN_2207291. ILMN_1721712 |

**Supplementary Table 4. Identification of genes selected from each cluster in the DAVID tools database.** Detailed list of genes from each cluster obtained from Supplementary Table 3.

| ID | Gene Name |
| --- | --- |
| *Enrichment Score: 1.295* | *Serine-type endopeptidase activity* |
| ILMN_1657446 | chromosome 1 open reading frame 57 |
| ILMN_1731233 | granzyme H (cathepsin G-like 2. protein h-CCPX) |
| ILMN_1797776 | protease. serine. 23 |
| ILMN_2053536 | rhomboid. veinlet-like 2 (Drosophila) |
|  |  |
| *Enrichment Score: 1.280* | *Apoptosis* |
| ILMN_1655577 | T-cell lymphoma invasion and metastasis 1 |
| ILMN_1697742 | chromosome 3 open reading frame 38 |
| ILMN_1731233 | granzyme H (cathepsin G-like 2. protein h-CCPX) |
| ILMN_1687440 | homeodomain interacting protein kinase 2 |
| ILMN_2207291 | interferon. gamma |
| ILMN_1811221 | solute carrier family 5 (iodide transporter). member 8 |
|  |  |
| *Enrichment Score: 0.936* | *Regulation of system process* |
| ILMN_1801216 | S100 calcium binding protein P |
| ILMN_2207291 | interferon. gamma |
| ILMN_1721712 | synaptogyrin 1 |

**Supplementary Table 5. Ingenuity database clusters.**

| *Top Diseases and Bio Functions* | | |
| --- | --- | --- |
| *Diseases and Disorders* | | |
| **Name** | ***P*-value** | **# Molecules** |
| Cancer | 1.66e-4 - 4.88e-2 | 26 |
| Reproductive System Disease | 1.66e-4 - 4.14e-2 | 15 |
| Inflammatory Response | 1.94e-4 - 4.83e-2 | 5 |
| Dermatological Diseases and Conditions | 2.33e-4 - 4.24e-2 | 9 |
| Inflammatory Diseases | 2.33e-4 - 4.53e-2 | 8 |
|  |  |  |
| *Molecular and cellular Functions* | | |
| **Name** | ***P*-value** | **# Molecules** |
| Drug Metabolism | 1.50e-5 - 4.24e-2 | 6 |
| Lipid Metabolism | 1.5e-5 - 4.83e-2 | 6 |
| Molecular Transport | 1.5e-5 - 4.83e-2 | 9 |
| Small Molecule Biochemistry | 1.5e-5 - 4.83e-2 | 10 |
| Cell Cycle | 6.39e-5 - 4.91e-2 | 13 |
|  |  |  |
| *Physiological System Development and Function* | | |
| **Name** | ***P*-value** | **# Molecules** |
| Hematological System Development and Function | 1.94e-4 - 4.83e-2 | 4 |
| Immune Cell Trafficking | 1.94e-4 - 4.83e-2 | 3 |
| Nervous System Development and Function | 1.05e-3 - 3.64e-2 | 3 |
| Hair and Skin Development and Function | 3.09e-3 - 1.84e-2 | 2 |
| Hematopoiesis | 3.09e-3 - 4.83e-2 | 3 |

**Supplementary Table 6: Histology score versus SLC5A8 expression for patients from the symptomatic cohort.** The infection status was classified as infected (1) or non-infected (0). Gastritis damage was scored as 0 (normal), 1 (mild), 2 (moderate) or 3 (severe). Inflammatory activity, lymphoid follicles and atrophy were scored as present (1) or absent (0). The histology score is a summary of histologic damage. The higher the score, the greater the gastric damage and inflammatory processes; the score is based on a scale of 0 (no damage), 1 (light damage), 2 (moderate damage) or 3 (severe damage).

|  | |  | |  | | | | | | | | | |
| --- | --- | --- | --- | --- | --- | --- | --- | --- | --- | --- | --- | --- | --- |
| *Samples* | | *Infected/non-infected* | | *Fold-changes* | *Gastritis* | *Inflammatory*  *Activity* | | | *Lymphoid follicles* | | *Atrophy* | | |
| 14 | | 0 | | 0.62 | 0 | 0 | | | 0 | | 0 | | |
| 20 | | 0 | | 1.81 | 0 | 0 | | | 0 | | 1 | | |
| 23 | | 0 | | 1.17 | NA | |  |  | |  | |  |  |
| 29 | | 0 | | 2.20 | 0 | 0 | | | 0 | | 0 | | |
| 39 | | 0 | | 3.64 | 1 | 0 | | | 0 | | 0 | | |
| 40 | | 0 | | 3.84 | 1 | 0 | | | 0 | | 0 | | |
| 42 | | 0 | | 4.08 | 0 | 0 | | | 0 | | 0 | | |
| 44 | | 0 | | 1.94 | 1 | 0 | | | 0 | | 0 | | |
| 47 | | 0 | | 0.19 | 1 | 0 | | | 0 | | 0 | | |
| 49 | | 0 | | 0.35 | 0 | 0 | | | 0 | | 0 | | |
| 52 | | 0 | | 2.06 | 0 | 0 | | | 0 | | 0 | | |
| 57 | | 0 | | 0.02 | 1 | 0 | | | 0 | | 0 | | |
| 58 | | 1 | | 0.02 | NA | | NA | NA | | NA | |  |  |
| 61 | | 1 | | 0.11 | 1 | 0 | | | 0 | | 0 | | |
| 63 | | 1 | | 0.29 | 1 | 1 | | | 0 | | 0 | | |
| 69 | | 1 | | 0.08 | 2 | 1 | | | 0 | | 0 | | |
| 73 | | 1 | | 0.05 | NA | | NA | NA | | NA | |  |  |
| 74 | | 1 | | 0.08 | NA | | NA | NA | | NA | |  |  |
| 78 | | 1 | | 0.07 | 2 | 1 | | | 0 | | 0 | | |
| 79 | | 1 | | 0.45 | 1 | 1 | | | 0 | | 0 | | |
| 80 | | 1 | | 0.46 | 1 | 0 | | | 0 | | 0 | | |
| 81 | | 1 | | 0.03 | 1 | 0 | | | 1 | | 0 | | |
| 83 | | 1 | | 0.11 | 2 | 1 | | | 0 | | 0 | | |
| 84 | | 1 | | 0.02 | 2 | 0 | | | 1 | | 0 | | |

NA: data no available

**Supplementary Table 7: Reported symptoms and corresponding SLC5A8 expression levels for children from the symptomatic cohort.** Symptoms reported included abdominal pain and vomiting; the severity of symptomatology was determined and scored as 0 (no symptoms), 1 (mild), 2 (moderate) or 3 (severe). The duration of symptoms is represented as 1 (last week), 2 (last month), 3 (last year).

| *Samples* | *Infected/non-infected* | *Fold-changes* | *Symptom Description* | *Symptom Duration* |
| --- | --- | --- | --- | --- |
| 61 | 0 | 0.62 | 3 | 1 |
| 14 | 0 | 1.81 | 2 | 3 |
| 23 | 0 | 1.17 | 3 | 2 |
| 29 | 0 | 2.20 | 1 | 3 |
| 44 | 0 | 3.64 | 1 | 2 |
| 52 | 0 | 3.84 | 2 | 1 |
| 57 | 0 | 4.08 | 3 | 1 |
| 58 | 0 | 1.94 | 2 | 1 |
| 63 | 0 | 0.19 | 2 | 1 |
| 69 | 0 | 0.35 | 2 | 1 |
| 73 | 0 | 2.06 | 1 | 1 |
| 74 | 0 | 0.02 | 1 | 2 |
| 20 | 1 | 0.02 | 3 | 3 |
| 39 | 1 | 0.11 | 3 | 1 |
| 40 | 1 | 0.29 | 3 | 2 |
| 42 | 1 | 0.08 | 3 | 3 |
| 47 | 1 | 0.05 | 2 | 2 |
| 49 | 1 | 0.08 | 1 | 2 |
| 78 | 1 | 0.07 | 2 | 1 |
| 79 | 1 | 0.45 | 2 | 1 |
| 80 | 1 | 0.46 | 3 | 1 |
| 81 | 1 | 0.03 | 2 | 1 |
| 83 | 1 | 0.11 | 0 | 0 |
| 84 | 1 | 0.02 | 3 | 2 |

**Supplementary Table 8. SLC5A8 expression data from the pilot study.** This table includes the values shown in Figure 3A. Here expression is represented as ΔCT, average CT β-actin and SL5A8 expression for infected and non-infected children. The Fold-change (FC) was determined using the median ΔCT of Infected versus non-infected children. The ΔΔCT was calculated as the difference between the ΔCT of non-infected versus infected children.

| INFECTED | | | |
| --- | --- | --- | --- |
| *Expression* | *ΔCT* | *CT*  *β-actin* | *CT*  *SLC5A8* |
| 0.083 | 3.59 | 20.65 | 24.24 |
| 0.001 | 9.44 | 20.14 | 29.58 |
| 0.369 | 1.44 | 21 | 22.44 |
| 1.E-04 | 13.11 | 20.362 | 33.47 |
| 0.912 | 0.13 | 20.051 | 20.18 |
| 2.E-10 | 30.00 | 19.5 | 49.50 |
| 4.E-09 | 25.00 | 19.6 | 44.60 |
| 0.026 | 5.28 | 20.682 | 25.96 |
| 0.042 | 4.58 | 21 | 25.58 |
|  |  |  |  |
| NON INFECTED | | | |
| *Expression* | *ΔCT* | *CT*  *β-actin* | *CT*  *SLC5A8* |
| 6.E-10 | 29.58 | 18.65 | 48.24 |
| 0.023 | 5.46 | 19.22 | 24.68 |
| 5.645 | -2.50 | 20.51 | 18.02 |
| 1.125 | -0.17 | 20.32 | 20.15 |
| 3.174 | -1.67 | 19.65 | 17.99 |
| 5.098 | -2.35 | 20.75 | 18.40 |
| 4.659 | -2.22 | 20.56 | 18.34 |
|  |  |  |  |
| FC Non-Infected Vs Infected | | | |
| *ΔCTSLC5A8 Median* | *ΔCTACTIN Median* | *ΔΔCT* | *FC* |
| 2,7 | -4,2 | 6,9 | 119 |

**Supplementary Table 9. SLC5A8 expression data from the case-control study.** This table includes the values shown in Figure 3B. Here expression is represented as ΔCT, average CT β-actin and SL5A8 expression for infected and non-infected children. The Fold-change (FC) was determined using the median ΔCT of Infected versus non-infected children. The ΔΔCT was calculated as the difference between the ΔCT of non-infected versus infected children. Expression levels were not detectable in samples from 12 infected and 4 non-infected children. ND: non-detectable. We assigned these samples an expression value of 0.

| INFECTED | | | |
| --- | --- | --- | --- |
| *Expression* | *ΔCT* | *CT*  *β-actin* | *CT*  *SLC5A8* |
| 18,12 | -4.18 | 20.35 | 16.17 |
| 0.00 | 30.40 | 19.22 | 49.62 |
| 0.00 | 28.92 | 20.51 | 49.43 |
| 5.31 | -2.41 | 20.32 | 17.91 |
| 0.47 | 1.09 | 19.65 | 20.74 |
| 0.00 |  | 20.75 | ND |
| 0.00 | 29.05 | 20.56 | 49.61 |
| 0.23 | 2.11 | 20.65 | 22.76 |
| 0.00 | 30.22 | 19.14 | 49.36 |
| 0.00 |  | 21.00 | ND |
| 0.00 | 30.01 | 19.36 | 49.37 |
| 9.68 | -3.28 | 20.05 | 16.78 |
| 2.44 | -1.29 | 19.50 | 18.21 |
| 0.28 | 1.84 | 19.60 | 21.44 |
| 5.71 | -2.51 | 20.68 | 18.17 |
| 0.10 | 3.28 | 21.00 | 24.28 |
| 0.00 |  | 18.65 | ND |
| 42.03 | -5.39 | 20.02 | 14.63 |
| 0.41 | 1.30 | 20.69 | 21.99 |
| 0.70 | 0.52 | 20.42 | 20.93 |
| 1.06 | -0.08 | 20.67 | 20.59 |
| 0.70 | 0.52 | 20.00 | 20.52 |
| 0.94 | 0.09 | 20.65 | 20.74 |
| 0.08 | 3.68 | 19.86 | 23.54 |
| 1.31 | -0.39 | 19.89 | 19.50 |
| 0.01 | 6.25 | 20.75 | 26.99 |
| 1.75 | -0.81 | 20.41 | 19.61 |
| 0.68 | 0.55 | 21.00 | 21.55 |
| 7.72 | -2.95 | 20.41 | 17.47 |
| 0.00 |  | 19.89 | ND |
| 0.00 |  | 19.62 | ND |
| 0.00 |  | 19.87 | ND |
| 18.69 | -4.22 | 20.75 | 16.52 |
| 0.00 |  | 20.15 | ND |
| 0.20 | 2.29 | 21.00 | 23.29 |
| 0.32 | 1.64 | 20.14 | 21.78 |
| 0.00 |  | 19.75 | ND |
| 0.00 |  | 19.55 | ND |
| 0.07 | 3.84 | 20.36 | 24.20 |
| 0.00 |  | 19.44 | ND |
| 0.00 | 13.25 | 20.33 | 33.58 |
| 6.54 | -2.71 | 20.14 | 17.43 |
| 3.30 | -1.72 | 20.00 | 18.28 |
| 0.00 |  | 19.63 | ND |
| 0.02 | 5.88 | 20.14 | 26.02 |
| 0.04 | 4.79 | 20.14 | 24.93 |
| 0.74 | 0.43 | 20.44 | 20.87 |
| 2.30 | -1.20 | 20.70 | 19.49 |
| 24.02 | -4.59 | 20.14 | 15.55 |
| 0.00 | 30.46 | 19.05 | 49.52 |
| 0.41 | 1.30 | 20.34 | 21.64 |
| 0.64 | 0.65 | 20.42 | 21.06 |
| 0.00 |  | 20.09 | ND |
| 0.01 | 7.57 | 20.32 | 27.89 |
|  |  |  |  |
| NON INFECTED | | | |
| *Expression* | *ΔCT* | *CT*  *β-actin* | *CT*  *SLC5A8* |
| 0.00 | 30.21 | 19.32 | 49.54 |
| 0.00 | 30.43 | 19.63 | 50.06 |
| 1.26 | -0.34 | 19.02 | 18.68 |
| 8.94 | -3.16 | 20.33 | 17.16 |
| 0.78 | 0.36 | 20.02 | 20.38 |
| 6.27 | -2.65 | 20.33 | 17.68 |
| 0.00 | 30.17 | 19.00 | 49.17 |
| 212.41 | -7.73 | 20.52 | 12.79 |
| 0.65 | 0.61 | 20.32 | 20.93 |
| 8.93 | -3.16 | 20.15 | 16.99 |
| 6.23 | -2.64 | 20.87 | 18.23 |
| 0.36 | 1.48 | 20.02 | 21.50 |
| 1.81 | -0.86 | 20.38 | 19.52 |
| 1.63 | -0.71 | 20.99 | 20.28 |
| 0.00 |  | 19.00 | ND |
| 1.50 | -0.58 | 20.63 | 20.05 |
| 3.77 | -1.92 | 19.63 | 17.72 |
| 0.87 | 0.20 | 20.97 | 21.17 |
| 19.03 | -4.25 | 20.30 | 16.05 |
| 18.47 | -4.21 | 20.33 | 16.13 |
| 5.88 | -2.56 | 20.42 | 17.86 |
| 2.92 | -1.55 | 20.30 | 18.75 |
| 12.74 | -3.67 | 19.05 | 15.37 |
| 0.00 | 14.31 | 19.02 | 33.33 |
| 4.87 | -2.28 | 20.89 | 18.61 |
| 31.04 | -4.96 | 20.90 | 15.94 |
| 2.54 | -1.34 | 19.63 | 18.28 |
| 0.00 |  | 19.32 | ND |
| 0.00 |  | 19.65 | ND |
| 1.63 | -0.71 | 20.66 | 19.95 |
| 0.00 |  | 19.89 | ND |
| 17.59 | -4.14 | 20.33 | 16.19 |
| 7.91 | -2.98 | 20.98 | 18.00 |
| 0.27 | 1.90 | 20.99 | 22.89 |
| 17.42 | -4.12 | 20.13 | 16.01 |
| 8.74 | -3.13 | 20.33 | 17.20 |
| 72.13 | -6.17 | 20.62 | 14.45 |
| 37.85 | -5.24 | 20.65 | 15.41 |
| 0.00 | 30.31 | 19.00 | 49.31 |
| 0.96 | 0.06 | 19.65 | 19.71 |
| 0.00 | 30.77 | 19.04 | 49.81 |
| 22.95 | -4.52 | 19.65 | 15.13 |
| 1.90 | -0.93 | 19.94 | 19.01 |
| 0.00 | 27.23 | 19.94 | 47.16 |
|  |  |  |  |
| *FC Non-Infected Vs Infected* | | | |
| *ΔCTActin Median* | *ΔCTSLC5A8 Median* | *ΔΔCT* | *FC* |
| 0,3 | 2,6 | 2,3 | 5 |

**Supplementary Table 10. Correlation between infection status, sero-positivity and mRNA SLC5A8 expression.** This table includes the values shown in Figure 3C. Here expression is represented as ΔCT, average CT β-actin and SL5A8 expression for infected and non-infected children. The Fold-change (FC) was determined using the median ΔCT of Infected versus non-infected children. The ΔΔCT was calculated as the difference between the ΔCT of non-infected versus infected children. Expression levels were not detectable in samples from 12 infected and 4 non-infected children. ND: non-detectable. We assigned these samples an expression value of 0.

| INFECTED/ SEROPOSITIVE | | | |
| --- | --- | --- | --- |
| *Expression* | *ΔCT* | *CT*  *β-actin* | *CT*  *SLC5A8* |
| 0.13 | 3.28 | 21.00 | 24.28 |
| 0.00 | 28.92 | 20.51 | 49.43 |
| 5.31 | -2.41 | 20.32 | 17.91 |
| 0.47 | 1.09 | 19.65 | 20.74 |
| 0.00 |  | 20.75 | ND |
| 0.00 | 29.05 | 20.56 | 49.61 |
| 0.00 | 30.22 | 19.14 | 49.36 |
| 0.00 |  | 21.00 | ND |
| 0.00 | 30.01 | 19.36 | 49.37 |
| 9.68 | -3.28 | 20.05 | 16.78 |
| 2.44 | -1.29 | 19.50 | 18.21 |
| 0.28 | 1.84 | 19.60 | 21.44 |
| 5.71 | -2.51 | 20.68 | 18.17 |
| 0.00 |  | 18.65 | ND |
| 0.70 | 0.52 | 20.42 | 20.93 |
| 0.94 | 0.09 | 20.65 | 20.74 |
| 0.08 | 3.68 | 19.86 | 23.54 |
| 7.72 | -2.95 | 20.41 | 17.47 |
| 0.00 |  | 19.89 | ND |
| 0.00 |  | 19.62 | ND |
| 0.00 |  | 19.87 | ND |
| 18.69 | -4.22 | 20.75 | 16.52 |
| 0.00 |  | 20.15 | ND |
| 0.20 | 2.29 | 21.00 | 23.29 |
| 0.00 |  | 19.75 | ND |
| 0.00 |  | 19.55 | ND |
| 0.07 | 3.84 | 20.36 | 24.20 |
| 0.00 |  | 19.44 | ND |
| 0.00 | 13.25 | 20.33 | 33.58 |
| 3.30 | -1.72 | 20.00 | 18.28 |
| 0.00 |  | 19.63 | ND |
| 0.02 | 5.88 | 20.14 | 26.02 |
| 0.74 | 0.43 | 20.44 | 20.87 |
| 2.30 | -1.20 | 20.70 | 19.49 |
| 24.02 | -4.59 | 20.14 | 15.55 |
| 0.00 | 30.46 | 19.05 | 49.52 |
| 0.41 | 1.30 | 20.34 | 21.64 |
| 0.64 | 0.65 | 20.42 | 21.06 |
| 0.00 |  | 20.09 | ND |
| 0.01 | 7.57 | 20.32 | 27.89 |
|  |  |  |  |
| NON INFECTED/ SERONEGATIVE | | | |
| *Expression* | *ΔCT* | *CT*  *β-actin* | *CT*  *SLC5A8* |
| 0.00 | 27.23 | 19.94 | 47.16 |
| 1.26 | -0.34 | 19.02 | 18.68 |
| 0.78 | 0.36 | 20.02 | 20.38 |
| 0.65 | 0.61 | 20.32 | 20.93 |
| 8.93 | -3.16 | 20.15 | 16.99 |
| 6.23 | -2.64 | 20.87 | 18.23 |
| 0.36 | 1.48 | 20.02 | 21.50 |
| 1.81 | -0.86 | 20.38 | 19.52 |
| 1.63 | -0.71 | 20.99 | 20.28 |
| 3.77 | -1.92 | 19.63 | 17.72 |
| 0.87 | 0.20 | 20.97 | 21.17 |
| 19.03 | -4.25 | 20.30 | 16.05 |
| 0.00 |  | 19.00 | ND |
| 2.92 | -1.55 | 20.30 | 18.75 |
| 12.74 | -3.67 | 19.05 | 15.37 |
| 0.00 | 14.31 | 19.02 | 33.33 |
| 4.87 | -2.28 | 20.89 | 18.61 |
| 31.04 | -4.96 | 20.90 | 15.94 |
| 2.54 | -1.34 | 19.63 | 18.28 |
| 0.00 |  | 19.32 | ND |
| 0.00 |  | 19.65 | ND |
| 17.59 | -4.14 | 20.33 | 16.19 |
| 0.27 | 1.90 | 20.99 | 22.89 |
| 17.42 | -4.12 | 20.13 | 16.01 |
| 8.74 | -3.13 | 20.33 | 17.20 |
| 37.85 | -5.24 | 20.65 | 15.41 |
| 0.00 |  | 19.00 | ND |
| 0.96 | 0.06 | 19.65 | 19.71 |
| 1.90 | -0.93 | 19.94 | 19.01 |
|  |  |  |  |
| FC Non-Infected/Seronegative vs Infected/Seropositive | | | |
| *ΔCTActin*  *Median* | *ΔCTSLC5A8 Median* | *ΔΔCT* | *FC* |
| 0,1 | 2,4 | 2,3 | 5 |

**Supplementary Table 11. Correlation between the virulence factor *cagA* and mRNA SLC5A8 expression.** This table includes the values shown in Figure 4C. Here expression is represented as ΔCT, average CT β-actin and SL5A8 expression for infected and non-infected children. The Fold-change (FC) was determined using the median ΔCT of Infected versus non-infected children. The ΔΔCT was calculated as the difference between the ΔCT of non-infected versus infected children.

| *cagA +* | | | |
| --- | --- | --- | --- |
| *Expression* | *ΔCT* | *CT*  *β-actin* | *CT*  *SLC5A8* |
| 0.00 | 28.92 | 20.51 | 49.4349704 |
| 0.47 | 1.09 | 19.65 | 20.7440735 |
| 0.00 |  | 20.75 | ND |
| 0.00 |  | 21.00 | ND |
| 0.00 | 30.01 | 19.36 | 49.3671561 |
| 9.68 | -3.28 | 20.05 | 16.7758588 |
| 2.44 | -1.29 | 19.50 | 18.2102098 |
| 0.28 | 1.84 | 19.60 | 21.4427503 |
| 0.00 |  | 18.65 | ND |
| 0.41 | 1.30 | 20.69 | 21.9917418 |
| 0.70 | 0.52 | 20.42 | 20.9335291 |
| 0.94 | 0.09 | 20.65 | 20.740952 |
| 0.08 | 3.68 | 19.86 | 23.5411905 |
| 1.75 | -0.81 | 20.41 | 19.6052836 |
| 7.72 | -2.95 | 20.41 | 17.4652499 |
| 0.00 |  | 19.89 | ND |
| 0.07 | 3.84 | 20.36 | 24.1989023 |
| 0.00 |  | 19.44 | ND |
| 0.02 | 5.88 | 20.14 | 26.0233309 |
| 0.41 | 1.30 | 20.34 | 21.6375322 |
|  |  |  |  |
| *cagA -* | | | |
| *Expression* | *ΔCT* | *CT*  *β-actin* | *CT*  *SLC5A8* |
| 0.000 | 30.40 | 19.22 | 49.62 |
| 5.310 | -2.41 | 20.32 | 17.91 |
| 0.000 | 30.22 | 19.14 | 49.36 |
| 5.706 | -2.51 | 20.68 | 18.17 |
| 0.103 | 3.28 | 21.00 | 24.28 |
| 1.057 | -0.08 | 20.67 | 20.59 |
| 0.697 | 0.52 | 20.00 | 20.52 |
| 0.013 | 6.25 | 20.75 | 26.99 |
| 0.683 | 0.55 | 21.00 | 21.55 |
| 0.000 |  | 19.89 | ND |
| 0.000 |  | 19.62 | ND |
| 0.000 |  | 19.87 | ND |
| 18.690 | -4.22 | 20.75 | 16.52 |
|  |  | 20.15 | ND |
| 0.204 | 2.29 | 21.00 | 23.29 |
| 0.321 | 1.64 | 20.14 | 21.78 |
| 0.000 |  | 19.75 | ND |
| 0.000 | 13.25 | 20.33 | 33.58 |
| 3.298 | -1.72 | 20.00 | 18.28 |
| 0.744 | 0.43 | 20.44 | 20.87 |
| 2.30 | -1.20 | 20.70 | 19.49 |
| 24.02 | -4.59 | 20.14 | 15.55 |
| 0.00 |  | 20.09 | ND |
| 0.01 | 7.57 | 20.32 | 27.89 |
|  |  |  |  |
| FC CagA- vs CagA+ | | | |
| *ΔCTACTIN Median* | *ΔCTSLC5A8*  *Median* | *ΔΔCT* | *FC* |
| 0,020 | 0,3 | 0,3 | 1 |

**SUPPLEMENTARY FIGURES**

**Supplementary Figure 1. Gastritis damage in tissue samples versus mRNA SLC5A8 expression.** The x-axis represents the severity of gastritis damage in the tissue samples. The scored is based on scale of 0 (normal), 1 (light damage), 2 (moderate damage) or 3 (severe damage). NA represents those samples for which there was no data available. There is a significant correlation between the severity of gastritis damage and low mRNA SLC5A8 expression (*P=*0.019).


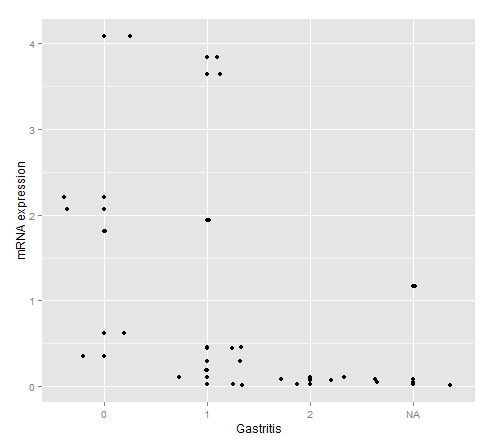


**Supplementary Figure 2. Lymphoid follicle damage in tissue samples versus mRNA SLC5A8 expression.** The x-axis represents the presence (1) or the absence (0) of lymphoid follicles in tissue samples. NA represents those samples for which there was no data available. There is a significant correlation between the presence of lymphoid follicles and low mRNA SLC5A8 expression (*P*=0.044).


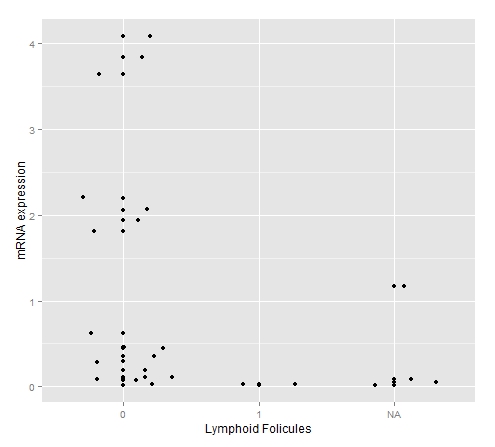


**Supplementary Figure 3. Activity of the inflammatory process in tissue samples versus mRNA SLC5A8 expression.** The x-axis represents the presence (1) or absence (0) of activity in the tissue sample. NA represents those samples for which there was no data available. There is no correlation between the activity of inflammatory process and mRNA SLC5A8 expression (*P*= 0.150).


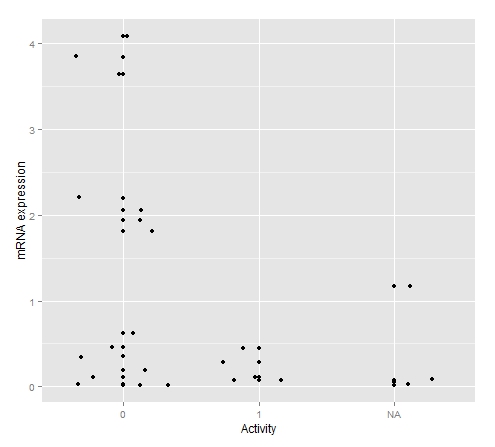


**Supplementary Figure 4. Presence of gastric atrophy in tissue samples versus mRNA SLC5A8 expression.** The x-axis represents the presence (1) or the absence (0) of atrophy in tissue samples. NA represents those samples for which there was no data available. There is no correlation between atrophy damage and mRNA SLC5A8 expression (*P*= 0.603).


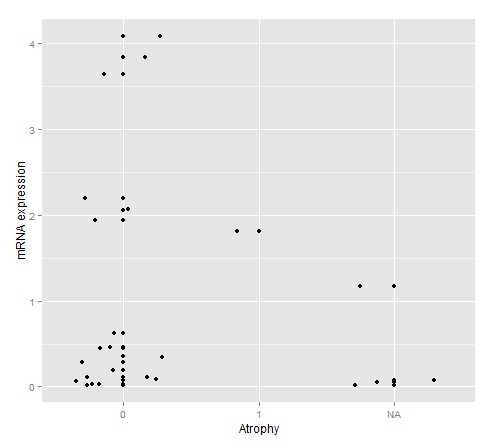


**Supplementary Figure 5. Severity of symptoms (vomiting and abdominal pain) versus mRNA SLC5A8 expression.** The x-axis represents the histology score, a metric of the severity of damage, in the tissue sample. The scored is based on scale of 0 (normal), 1 (light damage), 2 (moderate damage) or 3 (severe damage). NA represents those samples for which there was no data available. There is no correlation between symptomatology and mRNA SLC5A8 expression with a p= 0.872.


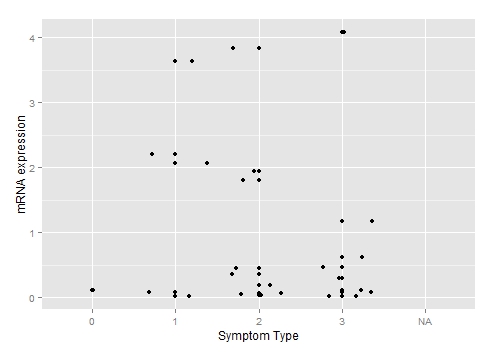

Supplement: Supplementary file 1 [file Table1.docx]
